# Inflammatory, antioxidant, and gut microbiota–related dietary indices and gastric cancer risk: a case–control study

**DOI:** 10.3389/fnut.2026.1893032

**Published:** 2026-09-16

**Authors:** Yongjing Chen, Rong Yang, Qingzhen Song, Yuan Li, Junjie Ma

**Affiliations:** 1 Department of Gastroenterology, Shanxi Cancer Hospital, Shanxi Hospital Affiliated to Cancer Hospital, Chinese Academy of Medical Sciences, Cancer Hospital Affiliated to Shanxi Medical University, Taiyuan, Shanxi, China; 2 Department of General Internal Medicine, Shanxi Cancer Hospital, Shanxi Hospital Affiliated to Cancer Hospital, Chinese Academy of Medical Sciences, Cancer Hospital Affiliated to Shanxi Medical University, Taiyuan, Shanxi, China; 3 Department of Gastrointestinal Surgery, Shanxi Cancer Hospital, Shanxi Hospital Affiliated to Cancer Hospital, Chinese Academy of Medical Sciences, Cancer Hospital Affiliated to Shanxi Medical University, Taiyuan, Shanxi, China

**Keywords:** gastric cancer, dietary inflammatory index, gut microbiota, oxidative stress, intestinal permeability

## Abstract

**Background:**

Dietary patterns may influence gastric carcinogenesis through inflammatory, oxidative, intestinal permeability, and gut microbiota-related pathways. However, comprehensive studies simultaneously evaluating inflammatory, antioxidant, and microbiota-oriented dietary indices together with multiple biological biomarkers in gastric cancer remain limited.

**Methods:**

This case–control study included 960 adults comprising 480 patients with histologically confirmed gastric cancer and 480 control participants. Dietary intake was assessed using a validated food frequency questionnaire. The Dietary Inflammatory Index (DII), Composite Dietary Antioxidant Index (CDAI), and Dietary Index for Gut Microbiota (DI-GM) were calculated and categorized into tertiles. Inflammatory, oxidative stress, intestinal permeability, and microbiota-related biomarkers were measured using standardized laboratory methods. Multivariable logistic regression analyses were performed to evaluate associations between dietary indices and gastric cancer risk.

**Results:**

Patients with gastric cancer had significantly higher DII scores and significantly lower CDAI and DI-GM scores compared with controls (all *p* < 0.001). In fully adjusted models, participants in the highest DII tertile had substantially greater odds of gastric cancer (OR: 6.21; 95% CI: 4.39–8.79), whereas higher CDAI (OR: 0.20; 95% CI: 0.14–0.28) and DI-GM (OR: 0.25; 95% CI: 0.18–0.35) were inversely associated with disease risk (all P-trend < 0.001). DII showed positive correlations with inflammatory and oxidative biomarkers, including CRP, TNF-α, MDA, zonulin, and NLR. However, the biomarker differences across dietary-index tertiles were generally modest. Among gastric cancer cases, DI-GM tertiles were significantly associated with zonulin concentrations. ROC analyses demonstrated moderate discriminatory performance for DII (AUC: 0.712), CDAI (AUC: 0.688), and DI-GM (AUC: 0.631).

**Conclusion:**

In conclusion, proinflammatory dietary patterns were associated with greater odds of gastric cancer, whereas diets rich in antioxidants or supportive of gut microbiota showed inverse associations. These associations were accompanied by differences in inflammatory, oxidative stress, intestinal permeability, and microbial metabolism related biomarkers. However, due to the case–control design, causality cannot be inferred, and the findings should be considered associational rather than causal.

## Introduction

Gastric cancer remains one of the leading causes of cancer-related morbidity and mortality worldwide and continues to impose a substantial public health burden, particularly in developing and transitional countries (1). Despite advances in diagnostic techniques and therapeutic strategies, prognosis remains poor for many patients because a considerable proportion of cases are diagnosed at advanced stages (2). Gastric carcinogenesis is a multifactorial and biologically complex process involving interactions among genetic susceptibility, chronic inflammation, microbial infection, environmental exposures, oxidative stress, and dietary habits (3). Among modifiable risk factors, nutrition has attracted increasing attention because dietary patterns may directly influence inflammatory signaling, mucosal integrity, oxidative balance, microbial composition, and tumor progression (4, 5).

Accumulating evidence suggests that pro-inflammatory dietary patterns characterized by high intake of processed foods, refined carbohydrates, saturated fats, salt-preserved foods, and ultra-processed products may contribute to gastric mucosal injury and carcinogenesis through activation of inflammatory and oxidative pathways (6, 7). Conversely, diets rich in fruits, vegetables, whole grains, antioxidants, dietary fiber, and polyphenols may exert protective effects by reducing oxidative stress, improving immune regulation, and supporting gut microbial homeostasis (8, 9). However, evaluating isolated nutrients may inadequately reflect the complexity of habitual dietary intake; therefore, comprehensive dietary indices have increasingly been used to characterize the cumulative biological effects of diet.

The Dietary Inflammatory Index (DII) is a validated tool designed to quantify the inflammatory potential of diet (10). Higher DII scores represent more pro-inflammatory dietary patterns and have been associated with elevated inflammatory biomarkers and increased risk of several chronic diseases and malignancies (11). In contrast, the Composite Dietary Antioxidant Index (CDAI) reflects cumulative antioxidant intake from major dietary antioxidants such as vitamins and trace minerals and may represent the antioxidative capacity of habitual diet (12). More recently, increasing attention has focused on the relationship between diet and gut microbiota in cancer development. The Dietary Index for Gut Microbiota (DI-GM) was developed to evaluate dietary patterns associated with favorable microbial composition and microbial metabolic activity (13).

Emerging evidence indicates that gastric carcinogenesis involves not only systemic inflammation but also oxidative DNA damage, intestinal barrier dysfunction, microbial dysbiosis, immune activation, and altered microbial metabolite production (14, 15). Nevertheless, important gaps remain in the current literature. Most previous studies on gastric cancer have evaluated inflammatory dietary patterns or antioxidant intake separately, while evidence regarding gut microbiota-related dietary indices remains very limited. Moreover, few investigations have simultaneously examined inflammatory, antioxidant, and microbiota-oriented dietary indices within the same gastric cancer population together with a comprehensive panel of inflammatory, oxidative stress, intestinal permeability, and microbial biomarkers. Therefore, the present study is one of the first to simultaneously evaluate DII, CDAI, and the emerging DIGM score within a single gastric cancer population, providing a comprehensive assessment of diet-related mechanisms involved in gastric carcinogenesis (16–18). Furthermore, prior investigations have commonly focused on a limited number of conventional inflammatory biomarkers such as C-reactive protein or interleukin-6, whereas comprehensive assessment of oxidative stress markers, gut permeability indicators, microbial metabolites, and intestinal inflammation-related biomarkers has been limited (8, 17).

Biomarkers including malondialdehyde (MDA), total antioxidant capacity (TAC), zonulin, lipopolysaccharide-binding protein (LBP), trimethylamine N-oxide (TMAO), fecal calprotectin, neutrophil-to-lymphocyte ratio (NLR), and 8-hydroxy-2′-deoxyguanosine (8-OHdG) may provide mechanistic insight into the biological pathways linking dietary exposures to gastric cancer risk and progression. However, few studies have comprehensively evaluated these biomarkers together in relation to inflammatory, antioxidant, and microbiota-oriented dietary indices. In addition, evidence regarding whether these associations differ according to tumor characteristics such as histological subtype, anatomical subsite, TNM stage, or metastatic status remains scarce and inconsistent (19). Previous studies have also frequently been limited by modest sample sizes, restricted biomarker profiling, or inadequate adjustment for major confounding factors including *Helicobacter pylori* infection, medication use, smoking, and lifestyle factors.

Therefore, the present case–control study aimed to comprehensively investigate the associations of DII, CDAI, and DI-GM with gastric cancer risk in adults. We additionally evaluated the relationships between these dietary indices and a broad panel of inflammatory, oxidative stress, intestinal permeability, nutritional, and microbiota-related biomarkers. Furthermore, we assessed the discriminatory performance of these dietary indices using LASSO and receiver operating characteristic analyses and explored whether observed associations differed according to tumor characteristics including histological type, anatomical location, disease stage, and metastatic status. Through integrating dietary pattern analysis with biomarker profiling and tumor-stratified analyses, this study sought to provide a more comprehensive understanding of the potential mechanistic links between diet, inflammation, gut dysfunction, microbial metabolism, and gastric carcinogenesis.

## Methods

### Study design and participants

This case–control study was conducted to investigate the associations of inflammatory, antioxidant, and gut microbiota-related dietary indices with gastric cancer risk and biomarker profiles among adults. The study included 960 participants, comprising 480 patients with histologically confirmed gastric cancer and 480 control participants without a history of malignancy. Cases were recruited from tertiary referral hospitals and oncology centers, while controls were selected from the same geographic regions through community-based recruitment and outpatient clinics.

### Sample size

Sample size calculations indicated that 480 cases and 480 controls provided 80% power to detect an odds ratio of 1.5 for a binary exposure with 30% prevalence in controls (*α* = 0.05, two-sided). Stratified analyses had reduced power due to smaller subgroup sizes; for example, a subgroup of 100 cases had only 40% power to detect an OR of 2.0. Stratified subgroup analyses were exploratory; no formal power calculations were performed for individual subgroups, and findings should be interpreted accordingly.

Eligible cases were adults aged ≥18 years with newly diagnosed gastric cancer confirmed by pathological examination. Participants with recurrent malignancy, previous chemotherapy or radiotherapy before enrollment, severe chronic inflammatory disorders, autoimmune disease, chronic renal failure, hepatic failure, or incomplete dietary data were excluded. To mitigate the potential for reverse causation, the FFQ assessed habitual dietary intake over the preceding year and specifically inquired about dietary patterns before the onset of gastrointestinal symptoms. Furthermore, participants who reported substantial dietary changes in the 6 months preceding diagnosis due to cancer related symptoms (e.g., early satiety, dysphagia, nausea, or anorexia) were excluded from analyses. Controls were frequency-matched to cases according to age and sex and were required to have no prior diagnosis of cancer or severe gastrointestinal disease. A total of 1,100 individuals were initially assessed for eligibility, comprising 567 eligible cases and 533 eligible controls. Of these, 87 cases and 53 controls were excluded due to incomplete FFQ data (defined as >10 missing food items or implausible total energy intake <500 or >5,000 kcal/day for women and <800 or >6,000 kcal/day for men). The final analytic sample comprised 480 cases and 480 controls, with an overall response rate of 87.3% (960/1,100). Cases were recruited from tertiary referral hospitals and oncology centers, while controls were selected from the same geographic regions through community-based recruitment and outpatient clinics (Figure 1, flowchart).

**Figure 1:**
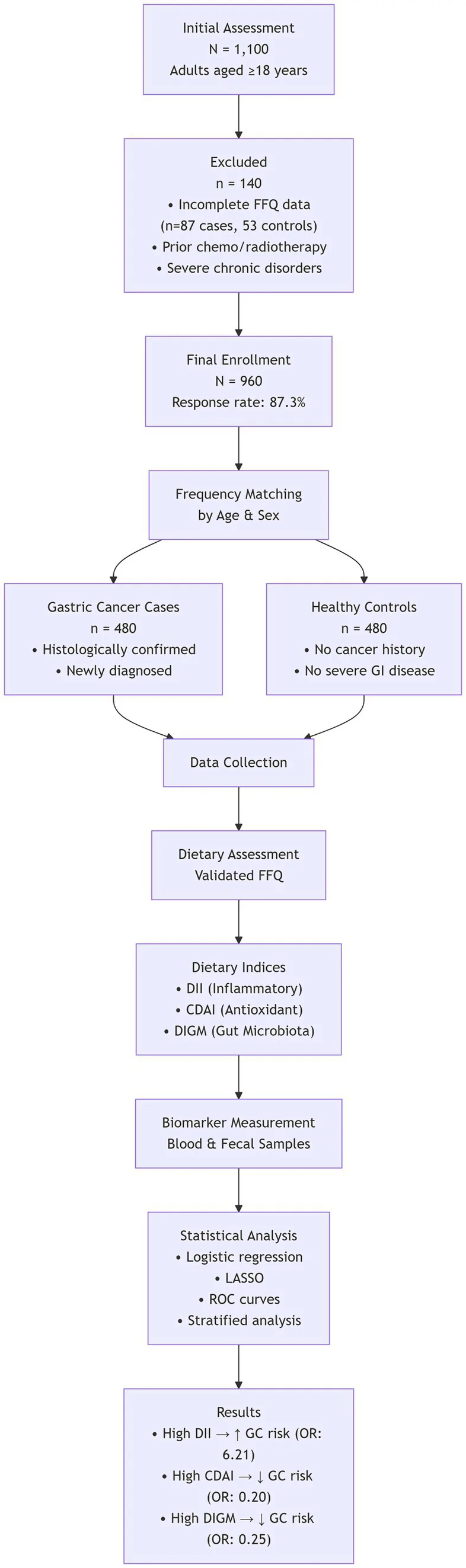
Study flowchart. Recruitment, exclusions, and final sample of 480 gastric cancer cases and 480 matched controls.

### Ethical considerations

The study protocol was approved by the Institutional Review Board of Shanxi Cancer Hospital (Approval No. IIT-2026-181). Written informed consent was obtained from all participants prior to enrollment. All study procedures were conducted in accordance with the ethical principles of the Declaration of Helsinki and relevant national regulations. Reporting of the present study was performed in accordance with the STROBE Initiative guidelines for observational studies, and a completed STROBE checklist was provided as Supplementary File 1.

### Clinical and tumor characteristics

Demographic and socioeconomic information including age, sex, marital status, educational level, occupation, socioeconomic status, and residential area were collected using structured interviewer-administered questionnaires. Lifestyle-related variables including smoking history, alcohol intake, physical activity, occupational exposure, NSAID use, and proton pump inhibitor (PPI)/H2 blocker use were also recorded.

Anthropometric assessment included measurement of body weight and height using standardized procedures, and body mass index (BMI) was calculated as weight in kilograms divided by height in meters squared (kg/m^2^).

Clinical information including *Helicobacter pylori* infection status, CagA/VacA positivity, Epstein–Barr virus (EBV) status, history of gastric surgery, atrophic gastritis, and family history of gastric cancer was obtained from medical records and participant interviews. Tumor-related variables among gastric cancer patients included histological subtype, anatomical tumor location, TNM stage, and metastatic status. Tumor characteristics were extracted from pathology reports at the time of diagnostic biopsy or surgical resection, prior to any neoadjuvant therapy.

### Dietary assessment

Habitual dietary intake was assessed using a validated semi-quantitative food frequency questionnaire (FFQ) administered by trained nutritionists. The FFQ had previously demonstrated acceptable validity and reproducibility in Chinese adults (20). Participants were asked to report the frequency and portion size of commonly consumed foods and beverages over the previous year. Daily nutrient intake was calculated using standard food composition databases.

Dietary variables included total energy intake, carbohydrate, protein, fat, total dietary fiber, soluble fiber, insoluble fiber, vitamin C, vitamin D, vitamin E, selenium, magnesium, salt intake, processed meat consumption, fermented food intake, coffee consumption, green tea intake, polyphenol intake, nitrate/nitrite intake, meal frequency, meal timing, and food preparation methods. Ultra-processed food consumption was evaluated using the NOVA classification system.

### Dietary indices

The DII was calculated according to the standardized method originally developed to quantify the inflammatory potential of habitual dietary intake. The DII was derived from dietary data obtained through the validated food frequency questionnaire (FFQ) and included available macro- and micronutrient components associated with inflammatory responses based on prior literature. Nutrient intakes were standardized using global reference values, transformed into centered percentile scores, and multiplied by corresponding inflammatory effect scores before summation to generate the overall DII score for each participant. Higher DII values indicated a more pro-inflammatory dietary pattern characterized by greater consumption of foods associated with systemic inflammation, whereas lower scores reflected more anti-inflammatory dietary habits (10).

The CDAI was calculated to estimate the cumulative antioxidant capacity of the habitual diet. The index incorporated standardized dietary intakes of major antioxidant nutrients, including vitamin C, vitamin E, vitamin D, selenium, magnesium, and other antioxidative micronutrients available from the FFQ dataset. Individual nutrient values were standardized using z-scores and combined to generate a composite antioxidant score. Higher CDAI values represented diets with greater antioxidant density and potentially enhanced capacity to counteract oxidative stress and free radical-mediated cellular damage (12).

The Dietary Index for Gut Microbiota (DI-GM) was calculated according to the method of Kase et al. (13), which includes 14 components: 10 beneficial items (fermented dairy products, chickpeas, soy products, whole grains, dietary fiber, cranberries, avocados, broccoli, coffee, and green tea) and four adverse items (red meat, processed meats, refined grains, and a high fat diet [≥40% of energy from fat]). Because DI-GM scoring is based on sex-specific median intake, it is less dependent on absolute energy intake. This index was originally developed and validated against measures of gut microbiota composition and metabolite production (e.g., short-chain fatty acids), demonstrating that higher DI-GM scores correlate with a more favorable gut microbial profile (15). For each component, sex-specific median intake levels were derived from the study population; beneficial components scored 1 point if intake was at or above the median (otherwise 0), while adverse components scored 1 point if intake was below the median (otherwise 0). The points were summed to yield a total DI-GM score ranging from 0 to 14, with higher scores indicating greater adherence to a gut microbiota-beneficial dietary pattern. The continuous score was used in regression analyses, and participants were also categorized into tertiles (T1–T3) based on the score distribution for descriptive comparisons (13).

For analytical purposes, DII, CDAI, and DI-GM scores were categorized into tertiles according to their distribution within the study population. The lowest tertile served as the reference category in regression analyses evaluating associations with gastric cancer risk and biomarker profiles.

### Laboratory and biomarker assessments

Fasting venous blood samples were obtained from all participants following an overnight fasting period of at least 10–12 h. Blood specimens were collected under standardized clinical conditions by trained laboratory personnel. Serum and plasma fractions were separated by centrifugation immediately after collection and stored at −80 °C until biochemical analysis to minimize biomarker degradation and analytical variability.

The present study evaluated a comprehensive panel of inflammatory, oxidative stress, intestinal permeability, microbiota-related, and nutritional biomarkers to provide a multidimensional assessment of biological pathways potentially linking diet with gastric carcinogenesis. Inflammatory biomarkers included C-reactive protein (CRP), interleukin-6 (IL-6), tumor necrosis factor-*α* (TNF-α), and neutrophil-to-lymphocyte ratio (NLR), which were selected because of their established roles in systemic inflammation, tumor progression, and immune activation.

Oxidative stress and antioxidant status were assessed using malondialdehyde (MDA), total antioxidant capacity (TAC), and 8-hydroxy-2′-deoxyguanosine (8-OHdG). MDA was evaluated as a marker of lipid peroxidation, TAC reflected cumulative antioxidant defense capacity, and 8-OHdG served as an indicator of oxidative DNA damage and cellular oxidative stress.

To investigate intestinal barrier dysfunction and microbiota-related metabolic alterations, several gut-associated biomarkers were measured, including zonulin, lipopolysaccharide-binding protein (LBP), TMAO, and calprotectin. Zonulin was assessed as a marker of intestinal permeability and epithelial tight-junction regulation, whereas LBP was included as an indicator of microbial translocation and endotoxin exposure, while TMAO reflected microbial-host co-metabolism associated with inflammatory and metabolic disturbances. Fecal calprotectin was measured as a marker of intestinal inflammation and mucosal immune activation.

Additional laboratory variables included serum albumin and red cell distribution width (RDW), which were evaluated as indicators of nutritional and systemic inflammatory status. Serum concentrations of C-reactive protein (CRP), interleukin-6 (IL-6), tumor necrosis factor-*α* (TNF-α), zonulin, lipopolysaccharide-binding protein (LBP), 8-hydroxy-2′-deoxyguanosine (8-OHdG), and trimethylamine N-oxide (TMAO) were quantified using commercially available enzyme-linked immunosorbent assay (ELISA) kits according to the manufacturers’ instructions. Malondialdehyde (MDA) and total antioxidant capacity (TAC) were measured using colorimetric assay kits. Intra- and inter-assay coefficients of variation were maintained below 10%. All assays were performed in duplicate under blinded laboratory conditions, following standardized procedures and the manufacturers’ quality-control recommendations, to minimize measurement bias and ensure analytical reliability.

### Statistical analysis

Statistical analyses were performed using Stata version 16 (StataCorp, College Station, TX, USA). Continuous variables were expressed as mean ± standard deviation (SD), while categorical variables were presented as frequency and percentage. Normality of data distribution was assessed using Q-Q plots, and the Shapiro–Wilk test. Comparisons between gastric cancer cases and controls were conducted using independent sample *t*-tests for continuous variables and chi-square tests for categorical variables. One-way analysis of variance (ANOVA) was used to compare biomarker levels across tertiles of dietary indices. Multivariable logistic regression models were used to evaluate associations between dietary index tertiles and gastric cancer risk. Odds ratios (ORs) and 95% confidence intervals (CIs) were estimated across tertiles using the lowest tertile as the reference category. Model 1 was adjusted for age and sex, while Model 2 was additionally adjusted for BMI, smoking pack-years, alcohol intake, physical activity, *H. pylori* infection, total energy intake, education level, medication use, socioeconomic status, and family history of gastric cancer. Tests for linear trend were performed by modeling tertile categories as ordinal variables.

Pearson correlation analyses were conducted to examine relationships between dietary indices and biomarker concentrations. LASSO regression analysis with 10-fold cross-validation was performed in two separate models: (1) a dietary/lifestyle-only model including DII, CDAI, DI-GM, age, sex, BMI, smoking, alcohol, physical activity, and energy intake to identify dietary predictors of gastric cancer status; and (2) an exploratory model including biomarkers (CRP, IL-6, TNF-α, MDA, TAC, zonulin, NLR) together with dietary indices. Results from model 2 should be interpreted cautiously due to potential overfitting and reverse causation, as biomarkers were measured after diagnosis. Receiver operating characteristic (ROC) curve analysis was performed to evaluate the predictive performance of DII, CDAI, and DI-GM, and area under the curve (AUC) values with 95% confidence intervals were calculated. Stratified analyses were additionally conducted according to histological subtype, anatomical tumor location, TNM stage, and metastatic status. Interaction analyses were performed by including multiplicative interaction terms in fully adjusted regression models. A two-sided *p* value <0.05 was considered statistically significant throughout all analyses. Given the large number of biomarker comparisons, Benjamini-Hochberg false discovery rate (FDR) correction was applied across the 42 pairwise tests in Supplementary Table 1, with *q* < 0.05 as the significance threshold. To address potential reverse causation, sensitivity analyses were performed excluding participants who reported gastrointestinal symptoms (early satiety, dysphagia, nausea, or anorexia) that may have led to dietary modifications prior to diagnosis. The results of these sensitivity analyses are presented in Supplementary Table 2.

## Results

### Participant characteristics

A total of 960 participants were included in the present case–control study, comprising 480 patients with gastric cancer and 480 control participants. Demographic, socioeconomic, anthropometric, lifestyle, and clinical characteristics of the study population are presented in Table 1. The mean age of participants was comparable between cases and controls (58.21 ± 10.72 vs. 58.32 ± 10.73 years, *p* = 0.869), and the proportion of men was identical in both groups (47.7%). No statistically significant differences were observed between groups regarding marital status, educational attainment, socioeconomic status, residential area, occupational exposure, smoking history, alcohol intake, physical activity, NSAID use, PPI/H2 blocker use, BMI, *Helicobacter pylori* infection, CagA/VacA positivity, family history of gastric cancer, history of gastric surgery, atrophic gastritis, EBV positivity, or total energy intake (all *p* > 0.05).

**Table 1:** Demographic, socioeconomic, lifestyle, anthropometric, and clinical characteristics of participants according to gastric cancer status.

Variable	Cases (*n* = 480)	Controls (*n* = 480)	*p*-value
Age (years), mean ± SD	58.21 ± 10.72	58.32 ± 10.73	0.869
Male sex, *n* (%)	229 (47.7)	229 (47.7)	1.000
Married status, *n* (%)	120 (25.0)	115 (24.0)	0.895
University education, *n* (%)	163 (34.0)	155 (32.3)	0.733
High socioeconomic status, *n* (%)	170 (35.4)	163 (34.0)	0.892
Urban residence, *n* (%)	231 (48.1)	243 (50.6)	0.439
Occupational exposure, *n* (%)	254 (52.9)	235 (49.0)	0.220
Smoking (pack-years), mean ± SD	13.04 ± 8.60	12.55 ± 7.90	0.361
Alcohol intake (g/day), mean ± SD	10.77 ± 6.59	10.54 ± 6.92	0.592
Regular physical activity, *n* (%)	238 (49.6)	250 (52.1)	0.439
NSAID use, *n* (%)	257 (53.5)	261 (54.4)	0.796
PPI/H2 blocker use, *n* (%)	254 (52.9)	236 (49.2)	0.245
Body mass index (kg/m^2^), mean ± SD	25.05 ± 3.92	25.16 ± 4.04	0.661
*H. pylori* positivity, *n* (%)	252 (52.5)	256 (53.3)	0.796
CagA/VacA positivity, *n* (%)	243 (50.6)	237 (49.4)	0.699
Family history of gastric cancer, *n* (%)	247 (51.5)	249 (51.9)	0.897
Atrophic gastritis, *n* (%)	225 (46.9)	225 (46.9)	1.000
History of gastric surgery, *n* (%)	240 (50.0)	256 (53.3)	0.301
EBV positivity, *n* (%)	46 (9.6)	58 (12.1)	0.213

SD, standard deviation; NSAID, nonsteroidal anti-inflammatory drug; PPI, proton pump inhibitor; H2 blocker, histamine-2 receptor antagonist; EBV, Epstein–Barr virus.

Values are mean ± SD for continuous variables and *n* (%) for categorical variables. Married status = category 1 of Marital Status; university education = category 3 of Education Level; high socioeconomic status = category 3 of Socioeconomic Status. *p*-values from independent t-test (continuous variables) or chi-square test (categorical variables).

### Dietary intake, food components, and dietary indices

Dietary characteristics and nutritional indices according to gastric cancer status are shown in Table 2. Patients with gastric cancer exhibited significantly higher DII scores compared with controls (2.66 ± 2.01 vs. 1.06 ± 2.04, *p* < 0.001), whereas CDAI and DI-GM scores were significantly lower among cases (15.08 ± 4.74 vs. 18.24 ± 4.49 and 5.01 ± 2.53 vs. 6.93 ± 2.97, respectively; both *p* < 0.001).

**Table 2:** Dietary intake variables, food components, and dietary indices according to gastric cancer status.

Variable	Cases (*n* = 480)	Controls (*n* = 480)	*p*-value
Dietary Inflammatory Index (DII)	2.66 ± 2.01	1.06 ± 2.04	<0.001
Composite Dietary Antioxidant Index (CDAI)	15.08 ± 4.74	18.24 ± 4.49	<0.001
Dietary Index for Gut Microbiota (DI-GM)	5.01 ± 2.53	6.93 ± 2.97	<0.001
Total energy intake (kcal/day), mean ± SD	2182.6 ± 443.7	2188.0 ± 441.3	0.850
Total carbohydrate intake (g/day)	281.6 ± 56.4	273.7 ± 59.6	0.035
Total protein intake (g/day)	81.3 ± 17.3	82.3 ± 17.8	0.376
Total fat intake (g/day)	75.8 ± 20.0	76.6 ± 19.2	0.507
Total dietary fiber (g/day)	21.94 ± 6.65	21.85 ± 6.72	0.835
Soluble fiber intake (g/day)	6.84 ± 2.14	6.96 ± 2.12	0.389
Insoluble fiber intake (g/day)	14.91 ± 3.92	15.01 ± 4.13	0.706
Vitamin C intake (mg/day)	86.3 ± 31.4	84.8 ± 29.2	0.450
Vitamin D intake (IU/day)	457.8 ± 175.7	464.9 ± 183.7	0.542
Vitamin E intake (mg/day)	11.94 ± 4.05	12.12 ± 3.93	0.486
Selenium intake (μg/day)	79.8 ± 20.1	80.9 ± 19.6	0.391
Magnesium intake (mg/day)	319.3 ± 61.3	320.9 ± 60.7	0.684
Salt intake (g/day)	8.90 ± 2.07	9.05 ± 1.96	0.257
Processed meat intake (servings/week)	4.01 ± 2.06	4.01 ± 2.03	0.997
Fermented food intake (servings/week)	2.96 ± 1.49	3.02 ± 1.49	0.522
Coffee intake (cups/day)	1.97 ± 1.04	2.03 ± 0.98	0.363
Green tea intake (cups/day)	1.47 ± 1.03	1.49 ± 1.00	0.834
Polyphenol intake (mg/day)	959 ± 211	939 ± 214	0.142
Nitrate/nitrite intake (mg/day)	119.2 ± 35.9	116.7 ± 35.6	0.269
Ultra-processed food score (NOVA)	2.79 ± 1.56	2.49 ± 1.13	<0.001
Fried/smoked food preparation, *n* (%)	279 (58.1)	167 (34.8)	<0.001

DII, Dietary Inflammatory Index; CDAI, Composite Dietary Antioxidant Index; DI-GM, Dietary Index for Gut Microbiota; NOVA, food classification system for ultra-processed foods (score 1–4).

Values are mean ± SD or *n* (%). *p*-values from independent t-test (continuous variables) or chi-square test (categorical variables).

Cases also demonstrated higher carbohydrate intake relative to controls (281.6 ± 56.4 vs. 273.7 ± 59.6 g/day, *p* = 0.035), although no significant differences were identified for total protein, total fat, dietary fiber, soluble fiber, insoluble fiber, vitamin C, vitamin D, vitamin E, selenium, magnesium, salt, processed meat intake, fermented food consumption, coffee intake, green tea intake, polyphenol intake, or nitrate/nitrite intake (all *p* > 0.05).

In contrast, notable differences were observed in dietary behavior and food processing characteristics. Participants with gastric cancer had significantly higher ultra-processed food (NOVA) scores (2.79 ± 1.56 vs. 2.49 ± 1.13, in Controls, *p* < 0.001). Cases also more frequently reported consuming foods prepared by frying or smoking (58.1% vs. 34.8%, p < 0.001).

### Biomarker profiles according to dietary index tertiles

Biomarker levels across tertiles of DII, CDAI, and DI-GM stratified by gastric cancer status are presented in Table 3. Among control participants, DII tertiles were associated with significantly lower IL-6 concentrations (*p* = 0.011), whereas no significant differences were observed for CRP, TNF-α, albumin, NLR, RDW, MDA, TAC, zonulin, 8-OHdG, LBP, TMAO, or fecal calprotectin across DII tertiles.

**Table 3:** Inflammatory, oxidative stress, gut barrier, nutritional, and microbiota-related biomarkers compared between gastric cancer cases and controls.

Biomarker	Cases (*n* = 480)	Controls (*n* = 480)	*p*-value
C-reactive protein (CRP; mg/L)	8.75 ± 2.66	5.65 ± 2.81	<0.001
Interleukin-6 (IL-6; pg./mL)	11.77 ± 3.95	8.37 ± 3.96	<0.001
Tumor necrosis factor-α (TNF-α; pg./mL)	14.18 ± 4.09	11.10 ± 4.09	<0.001
Albumin (g/dL)	3.98 ± 0.51	3.98 ± 0.50	0.915
Neutrophil-to-lymphocyte ratio (NLR)	3.80 ± 1.30	2.80 ± 1.24	<0.001
Red cell distribution width (RDW; %)	13.58 ± 1.15	13.51 ± 1.18	0.386
Malondialdehyde (MDA; μmol/L)	4.77 ± 1.20	3.82 ± 1.25	<0.001
Total antioxidant capacity (TAC; mmol/L)	1.11 ± 0.31	1.31 ± 0.30	<0.001
Zonulin (ng/mL)	56.39 ± 11.89	48.10 ± 12.12	<0.001
8-OHdG (ng/mL)	5.99 ± 1.92	5.93 ± 1.88	0.617
Lipopolysaccharide-binding protein (LBP; μg/mL)	18.26 ± 5.89	18.08 ± 6.32	0.659
Trimethylamine N-oxide (TMAO; μmol/L)	4.54 ± 1.44	4.50 ± 1.52	0.670
Fecal calprotectin (μg/g)	54.52 ± 19.28	54.14 ± 17.74	0.751

CRP, C-reactive protein; IL-6, interleukin-6; TNF-α, tumor necrosis factor-α; NLR, neutrophil-to-lymphocyte ratio; RDW, red cell distribution width; MDA, malondialdehyde; TAC, total antioxidant capacity; 8-OHdG, 8-hydroxy-2′-deoxyguanosine; LBP, lipopolysaccharide-binding protein; TMAO, trimethylamine N-oxide.

Values are mean ± SD. *p*-values from independent *t*-test.

Across CDAI tertiles, no statistically significant trends were identified for inflammatory, oxidative stress, gut permeability, or microbiota-related biomarkers in either controls or gastric cancer cases (all *p* > 0.05). Similarly, most biomarkers did not differ significantly across DI-GM tertiles in either group.

Among gastric cancer cases, DI-GM tertiles showed nominal associations with zonulin (*p* = 0.021) in uncorrected analyses. However, after applying Benjamini–Hochberg false discovery rate (FDR) correction for the 42 comparisons in Supplementary Table 1, none of these associations remained statistically significant (all *q* > 0.05). These biomarker findings should therefore be interpreted as exploratory and hypothesis generating, rather than as confirmatory evidence of pathway specific effects. The primary confirmatory findings of this study are the robust associations between the three dietary indices and gastric cancer risk, which were prespecified and adequately powered (Table 4).

**Table 4:** Multivariable logistic regression analysis of associations between dietary indices and gastric cancer risk: Tertile and continuous models.

Dietary index	Comparison	Crude OR (95% CI)	Model 1 OR (95% CI)*	Model 2 OR (95% CI)†	P-trend (Model 2)
DII					
	T1 (lowest)	1.00 (ref)	1.00 (ref)	1.00 (ref)	
	T2	2.32 (1.67–3.21)	2.32 (1.68–3.22)	2.33 (1.68–3.24)	
	T3 (highest)	6.09 (4.32–8.58)	6.12 (4.34–8.62)	6.21 (4.39–8.79)	**<0.001**
	Per 1-unit increase	–	–	**1.49 (1.39–1.61)**	**<0.001**
CDAI					
	T1 (lowest)	1.00 (ref)	1.00 (ref)	1.00 (ref)	
	T2	0.43 (0.31–0.59)	0.42 (0.31–0.59)	0.42 (0.30–0.58)	
	T3 (highest)	0.20 (0.15–0.28)	0.20 (0.15–0.28)	0.20 (0.14–0.28)	**<0.001**
	Per 1-unit increase	–	–	**0.86 (0.84–0.89)**	**<0.001**
DI-GM					
	T1 (lowest)	1.00 (ref)	1.00 (ref)	1.00 (ref)	
	T2	0.66 (0.48–0.90)	0.66 (0.48–0.90)	0.67 (0.48–0.92)	
	T3 (highest)	0.25 (0.18–0.35)	0.25 (0.18–0.35)	0.25 (0.18–0.35)	**<0.001**
	Per 1-unit increase	–	–	**0.77 (0.73–0.81)**	**<0.001**

Indices and gastric cancer risk – tertile and continuous models.

*Model 1: Adjusted for age and sex.

†Model 2: Additionally adjusted for BMI, smoking pack-years, alcohol intake, physical activity, *H. pylori* status, total energy intake, education level, PPI/H2 blocker use, socioeconomic status, and family history of gastric cancer.

P-trend: Calculated across tertiles using Model 2. The continuous per-unit increase rows are from Model 2 only (linear effect).

Bold values indicate statistical significance (*P* < 0.05).

Supplementary Table 1 further summarizes the distribution of inflammatory, oxidative stress, intestinal permeability, and microbiota-related biomarkers across tertiles of dietary indices stratified by case–control status. Overall, the majority of biomarkers showed modest variation across dietary index categories, although exploratory analyses suggested nominal associations between DI-GM and zonulin among gastric cancer patients.

### Multivariable associations between dietary indices and gastric Cancer risk

Multivariable logistic regression analyses examining the associations between dietary indices and gastric cancer risk are presented in Table 4 and Figure 2. Higher DII scores were strongly associated with greater odds of gastric cancer. Compared with participants in the lowest tertile, those in the highest tertile of DII had more than six-fold greater odds of gastric cancer in the fully adjusted model (OR: 6.21, 95% CI: 4.39–8.79; *P*-trend < 0.001).

**Figure 2:**
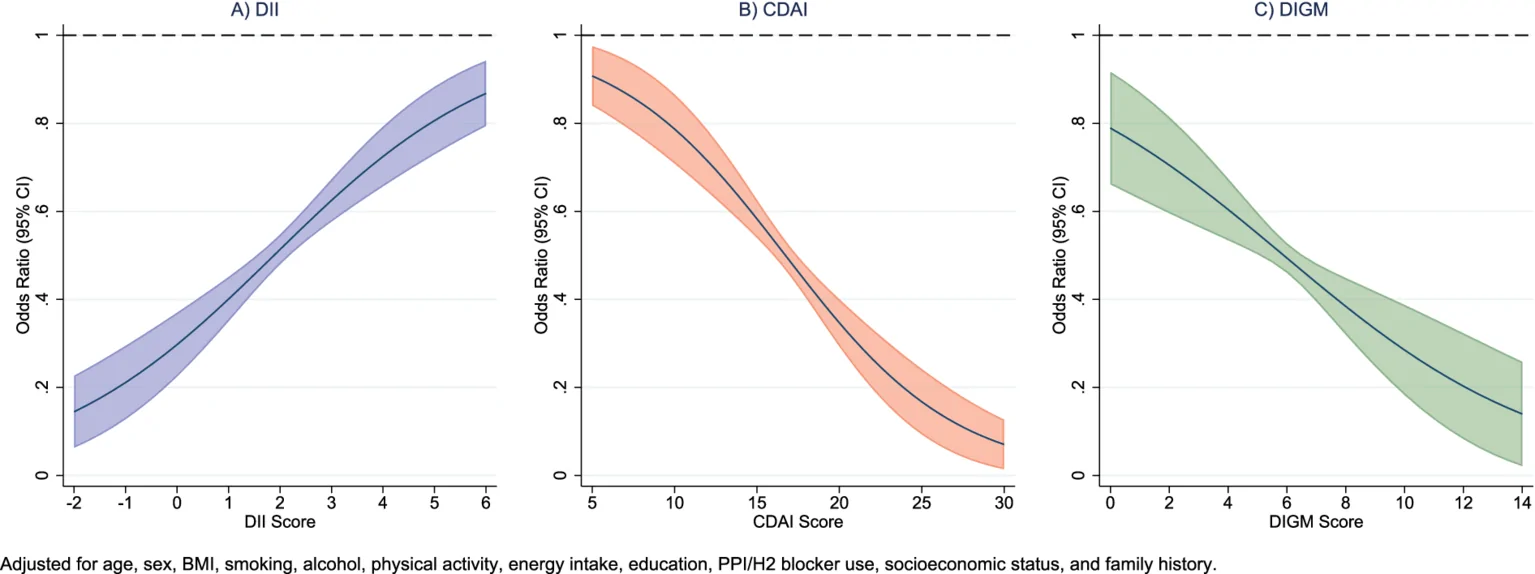
Dose–response associations of DII, CDAI, and DI-GM with gastric cancer risk. Adjusted restricted cubic spline plots (solid lines = OR, shaded areas = 95% CI). Higher DII increases risk; higher CDAI/DI-GM decrease risk (all P-trend < 0.001). Histograms show index distributions.

Conversely, higher CDAI scores were inversely associated with gastric cancer risk. Participants in the highest CDAI tertile demonstrated an approximately 80% lower likelihood of gastric cancer compared with those in the lowest tertile (OR: 0.20, 95% CI: 0.14–0.28; *P*-trend < 0.001). Similarly, higher DI-GM scores were significantly associated with reduced gastric cancer risk, with participants in the highest tertile exhibiting 75% lower odds of disease compared with the reference tertile (OR: 0.25, 95% CI: 0.18–0.35; *P*-trend < 0.001). These associations remained robust after adjustment for demographic variables, BMI, smoking, alcohol intake, physical activity, *H. pylori* infection, total energy intake, education level, medication use, socioeconomic status, and family history of gastric cancer.

In sensitivity analyses excluding participants who reported gastrointestinal symptoms (early satiety, dysphagia, nausea, or anorexia) that may have led to dietary modifications prior to diagnosis (*n* = 237 cases, 49.4% of the case group), the associations remained consistent with the main findings (Supplementary Table 2). These sensitivity analyses suggest that the observed associations are unlikely to be entirely attributable to reverse causation.

### Correlation, LASSO, and ROC analyses

Integrated correlation, penalized regression, and the ROC analyses are presented in Table 5. Pearson correlation analysis demonstrated that DII was positively correlated with inflammatory and oxidative stress biomarkers, including CRP (*r* = 0.15), IL-6 (*r* = 0.07), TNF-*α* (*r* = 0.12), MDA (*r* = 0.12), zonulin (*r* = 0.12), and NLR (*r* = 0.14) (all *p* < 0.05). In contrast, CDAI generally exhibited inverse correlations with inflammatory biomarkers, whereas DI-GM demonstrated inverse correlations with CRP, IL-6, TNF-α, MDA, and zonulin.

**Table 5:** Correlation, LASSO, and ROC analyses of dietary indices and biomarkers.

A: Pearson correlation matrix									
Variable	DII	CDAI	DI-GM	CRP	IL-6	TNF-α	MDA	TAC	Zonulin
DII	1.00								
CDAI	−0.10*	1.00							
DI-GM	−0.29*	0.05	1.00						
CRP	0.15*	−0.17*	−0.06	1.00					
IL-6	0.07*	−0.12*	−0.10*	0.19*	1.00				
TNF-α	0.12*	−0.09*	−0.07*	0.13*	0.12*	1.00			
MDA	0.12*	−0.15*	−0.08*	0.16*	0.15*	0.15*	1.00		
TAC	−0.13*	0.05	0.07*	−0.07*	−0.16*	−0.10*	−0.10*	1.00	
Zonulin	0.12*	−0.10*	−0.07*	0.16*	0.10*	0.11*	0.10*	−0.05	1.00
NLR	0.14*	−0.10*	−0.07*	0.16*	0.20*	0.16*	0.13*	−0.12*	0.11*

B: LASSO regression	
Variable	Coefficient
DII	0.837
CDAI	−0.670
DI-GM	−0.773
CRP	1.453
IL-6	1.052
TNF-α	0.874
Albumin	0.029
NLR	0.805
RDW	0.072
MDA	0.722
TAC	−0.724
Zonulin	0.843

C: ROC analysis		
Index	AUC	95% CI
DII	0.712	0.680–0.744
CDAI	0.688	0.655–0.722
DI-GM	0.631	0.596–0.666

*p < 0.05.

DII, Dietary Inflammatory Index; CDAI, Composite Dietary Antioxidant Index; DI-GM, Dietary Index for Gut Microbiota; CRP, C-reactive protein; IL-6, interleukin-6; TNF-α, tumor necrosis factor-α; MDA, malondialdehyde; TAC, total antioxidant capacity; NLR, neutrophil-to-lymphocyte ratio; RDW, red cell distribution width; LASSO, least absolute shrinkage and selection operator; ROC, receiver operating characteristic; AUC, area under the curve; CI, confidence interval.

In panel A: *p < 0.05. Pearson correlation coefficients were calculated across all participants.

In panel B: LASSO logistic regression was performed with 10-fold cross-validation. The tuning parameter λ (lambda) was selected as the value that gave the minimum mean cross-validated deviance (λ = 0.0021425). Only active variables with non-zero coefficients are shown. Positive coefficients indicate increased risk of gastric cancer, while negative coefficients indicate decreased risk.

In panel C: AUC values >0.5 indicate positive discrimination (higher index scores associated with higher probability of being a case). For CDAI and DI-GM, higher scores were inversely associated with gastric cancer risk; the reported AUCs (0.688 and 0.631, respectively) reflect the discrimination of the index for case status in the direction of higher scores → lower risk. No transformation to 1-AUC is needed or appropriate. All models were adjusted for age, sex, BMI, smoking pack-years, alcohol intake, physical activity, total energy intake, education level, socioeconomic status, and family history of gastric cancer.

LASSO regression identified several variables with the strongest contributions to gastric cancer discrimination, including CRP (coefficient = 1.453), IL-6 (1.052), TNF-α (0.874), zonulin (0.843), DII (0.837), NLR (0.805), and MDA (0.722). Negative coefficients were observed for CDAI (−0.670), DI-GM (−0.773), and TAC (−0.724), suggesting potential protective associations. The exploratory LASSO findings should be interpreted cautiously because biomarkers were measured after diagnosis and the model was not externally validated. Note that correlation analyses (Table 5) reflect continuous linear associations across all participants, whereas tertile-based ANOVA examines mean differences between discrete groups and is therefore less sensitive; both approaches are complementary.

ROC analysis demonstrated modest discriminatory performance for DII (AUC = 0.712, 95% CI: 0.680–0.744), CDAI (AUC = 0.688, 95% CI: 0.655–0.722), and DI-GM (AUC = 0.631, 95% CI: 0.596–0.666).

### Stratified analyses according to tumor characteristics

Stratified analyses evaluating associations between dietary indices and gastric cancer according to tumor characteristics are shown in Table 6. Elevated DII scores were consistently associated with higher gastric cancer risk across histological subtypes, anatomical subsites, TNM stages, and metastasis categories. The strongest association was observed among patients with stage IV disease (OR: 3.49, 95% CI: 2.17–5.61).

**Table 6:** Stratified associations of dietary indices with gastric cancer risk according to tumor characteristics.

Variable	Total cases (*n* = 480)	DII T3 vs T1 OR (95% CI)	CDAI T3 vs T1 OR (95% CI)	DI-GM T3 vs T1 OR (95% CI)	P-interaction	P-comparison among indices
Histological type						
Intestinal	165 (34.4)	2.61 (1.74–3.93)	0.63 (0.41–0.97)	0.49 (0.31–0.77)	0.184	0.021
Diffuse	156 (32.5)	2.97 (1.88–4.68)	0.55 (0.33–0.89)	0.39 (0.24–0.64)	0.093	0.008
Mixed	159 (33.1)	2.48 (1.61–3.81)	0.71 (0.45–1.12)	0.56 (0.35–0.89)	0.271	0.067
Anatomical subsite						
Cardia	251 (52.3)	2.89 (1.94–4.30)	0.68 (0.45–1.02)	0.52 (0.33–0.81)	0.146	0.018
Non-cardia	229 (47.7)	2.73 (1.81–4.12)	0.57 (0.37–0.88)	0.41 (0.26–0.66)	0.111	0.011
TNM stage						
Stage I	121 (25.2)	1.88 (1.12–3.14)	0.81 (0.49–1.34)	0.69 (0.40–1.17)	0.384	0.119
Stage II	117 (24.4)	2.34 (1.45–3.79)	0.67 (0.40–1.09)	0.53 (0.31–0.89)	0.209	0.042
Stage III	123 (25.6)	3.08 (1.94–4.88)	0.56 (0.34–0.92)	0.41 (0.24–0.69)	0.074	0.006
Stage IV	119 (24.8)	3.49 (2.17–5.61)	0.49 (0.29–0.83)	0.34 (0.20–0.58)	0.031	0.002
Metastasis status						
Positive	237 (49.4)	3.26 (2.08–5.09)	0.52 (0.32–0.84)	0.37 (0.22–0.61)	0.049	0.003
Negative	243 (50.6)	2.41 (1.58–3.67)	0.69 (0.45–1.05)	0.57 (0.36–0.90)	0.188	0.036

DII, Dietary Inflammatory Index; CDAI, Composite Dietary Antioxidant Index; DI-GM, Dietary Index for Gut Microbiota; T3, highest tertile; T1, lowest tertile; OR, odds ratio; CI, confidence interval; TNM, tumor-node-metastasis.

P-interaction values were calculated using multiplicative interaction terms in fully adjusted models. P-comparison values represent heterogeneity across dietary indices.

All stratified models were adjusted for the same covariates as Model 2 in Table 4: age, sex, BMI, smoking pack-years, alcohol intake, physical activity, *H. pylori* status, total energy intake, education level, PPI/H2 blocker use, socioeconomic status, and family history of gastric cancer. Models were not adjusted for tumor characteristics.

In contrast, higher CDAI and DI-GM scores were consistently associated with lower gastric cancer risk across most tumor strata. The inverse associations appeared strongest among diffuse-type tumors, advanced-stage disease, and metastatic cancers. For example, the highest DI-GM tertile was associated with substantially reduced odds of metastatic gastric cancer (OR: 0.37, 95% CI: 0.22–0.61).

No statistically significant interactions were observed between dietary indices and tumor subgroups (all *P*-interaction > 0.05), indicating that the observed associations were generally consistent across tumor characteristics. The odds ratios for DII in stratified analyses (range: 1.88–3.49) were lower than in the main analysis (OR = 6.21). This discrepancy likely reflects the smaller sample sizes in each stratum, adjustment for the same set of covariates (age, sex, BMI, smoking, alcohol, physical activity, *H. pylori* status, total energy intake, education, PPI use, socioeconomic status, and family history), and possible effect modification by tumor characteristics, though interaction tests were not statistically significant. These discrepancies do not represent analytical inconsistency but illustrate the importance of adequately powered subgroup analyses in future studies.

## Discussion

In this large case–control study, we comprehensively evaluated the associations of three biologically relevant dietary indices—the DII, CDAI, and DI-GM—with gastric cancer risk, inflammatory and oxidative biomarkers, intestinal permeability markers, and microbiota-related metabolites. Several important findings emerged from the present investigation. First, patients with gastric cancer exhibited significantly higher DII scores and significantly lower CDAI and DI-GM scores compared with controls, suggesting that proinflammatory dietary patterns and reduced antioxidant and microbiota supportive dietary quality may be associated with gastric carcinogenesis. However, given the case–control design, these associations should not be interpreted as causal. Second, higher DII scores were strongly associated with greater odds of gastric cancer, whereas higher CDAI and DI-GM scores demonstrated robust inverse associations with disease risk even after adjustment for confounders. Third, dietary indices showed meaningful correlations with systemic inflammatory, oxidative stress, and gut-related biomarkers. Finally, the observed associations remained relatively consistent across tumor subtypes, anatomical locations, disease stages, and metastatic status, suggesting that the potential biological effects of diet may extend across heterogeneous gastric cancer phenotypes.

Chronic gastric mucosal inflammation induced by dietary salt, nitrosamine exposure, processed meat intake, and ultra-processed foods may enhance epithelial injury, promote oxidative DNA damage, and facilitate progression from chronic gastritis to intestinal metaplasia and gastric carcinoma. Moreover, pro-inflammatory dietary patterns may amplify *Helicobacter pylori*-associated inflammatory signaling through activation of NF-κB, IL-6, and TNF-α pathways, thereby contributing to tumor-promoting microenvironmental changes (15, 18, 21). In the present study, participants with gastric cancer demonstrated substantially higher ultra-processed food intake and greater consumption of fried or smoked foods, further supporting the role of dietary inflammatory exposure in tumor development. The positive correlations between DII and CRP, IL-6, TNF-α, MDA, zonulin, and NLR reinforce the hypothesis that inflammatory dietary patterns may contribute to systemic immune activation, oxidative injury, and intestinal barrier dysfunction.

The present findings regarding the positive association between higher DII scores and gastric cancer are generally consistent with previous epidemiological investigations. A meta-analysis by Du et al. (18) reported a pooled OR of 2.11 (95% CI: 1.41–3.15) for the association between DII and gastric cancer risk, while another meta-analysis found that individuals in the highest DII category had an increased risk of upper gastrointestinal cancer (OR = 1.81, 95% CI: 1.65–1.97). The larger effect size observed in our study (OR = 6.21 for the highest vs. lowest tertile) may reflect several factors: (1) our study population from Shanxi Province represents a high-risk region for gastric cancer with distinct dietary patterns characterized by high consumption of salt-preserved and ultra-processed foods; (2) the fully adjusted model incorporated a more comprehensive set of confounders than many previous studies; (3) tertile categorization may yield larger contrasts than continuous or quartile-based approaches; and (4) population-specific genetic susceptibility may amplify the effect. Notably, stratified analyses yielded more moderate ORs (range: 1.88–3.49), consistent with published estimates and likely reflecting smaller stratum sample sizes. Furthermore, sensitivity analyses excluding participants with gastrointestinal symptoms (*n* = 237 cases, 49.4%) yielded an OR of 5.61 (95% CI: 3.60–8.73), which remains higher than previous meta-analyses but suggests that reverse causation does not entirely explain the magnitude of the association. Thus, the main analysis OR of 6.21 should be interpreted with appropriate caution and considered hypothesis-generating rather than definitive. Prior studies evaluating DII specifically have similarly demonstrated positive associations between higher inflammatory dietary scores and gastrointestinal malignancies, including gastric cancer. The current study extends previous evidence by demonstrating that the association between DII and gastric cancer remained robust after adjustment for major confounding factors, including *Helicobacter pylori* infection, smoking, BMI, medication use, and socioeconomic variables. Furthermore, unlike many earlier studies that evaluated only conventional inflammatory markers, the present investigation additionally incorporated oxidative stress, intestinal permeability, and microbiota-related biomarkers to provide a broader biological characterization of inflammatory dietary patterns in gastric cancer (22, 23). It is essential to interpret these associations with caution given the case–control design. Individuals with undiagnosed gastric cancer may have altered their dietary habits due to early symptoms such as early satiety, dysphagia, nausea, or anorexia, potentially biasing estimates of the diet–cancer association (24). This phenomenon—whereby disease-related symptoms influence dietary intake before diagnosis rather than diet influencing disease risk—represents a form of reverse causation inherent to case–control studies. To mitigate this concern, the FFQ assessed habitual dietary intake over the preceding year and explicitly inquired about dietary patterns before symptom onset. Furthermore, sensitivity analyses excluding participants who reported gastrointestinal symptoms that may have led to dietary modifications prior to diagnosis (*n* = 237 cases, 49.4% of the case group) yielded consistent results, with the direction and magnitude of associations remaining largely unchanged Supplementary Table 1. These sensitivity analyses suggest that the observed associations are unlikely to be entirely attributable to reverse causation. Despite these efforts, residual reverse causation and differential recall between cases and controls cannot be fully excluded. Prospective cohort studies with repeated dietary assessments are therefore needed to establish temporal relationships and strengthen causal inference. With this important caveat, the strong and consistent associations observed suggest that dietary patterns may be associated with gastric carcinogenesis, but these findings should be considered associational rather than causal.

Conversely, higher CDAI scores were associated with markedly lower odds of gastric cancer. One possible explanation for the observed association is that antioxidant-rich diets may reduce oxidative DNA damage and inflammatory signaling, although this mechanism cannot be established in the present case–control study. Modulation of inflammatory signaling, scavenging of reactive oxygen species, and preservation of cellular antioxidant defenses (25, 26). Gastric mucosal tissue is particularly vulnerable to oxidative stress because chronic inflammation, microbial infection, and environmental exposures can induce persistent free radical generation (27). Antioxidant nutrients including vitamins C and E, selenium, magnesium, and polyphenols may therefore exert protective effects by reducing lipid peroxidation and oxidative genomic injury (28). Although associations between CDAI tertiles and most biomarkers were not statistically significant in subgroup analyses, inverse correlations between CDAI and inflammatory markers suggest that antioxidant dietary quality may still influence systemic inflammatory homeostasis. The relatively modest biomarker differences across CDAI tertiles may reflect the multifactorial nature of oxidative regulation in gastric cancer or residual biological heterogeneity among participants.

An additional major finding of the present study was the inverse association between DI-GM and gastric cancer risk. Increasing evidence suggests that gut microbiota dysbiosis, altered microbial metabolite production, and intestinal barrier dysfunction contribute substantially to gastric and gastrointestinal carcinogenesis (15). Diet represents one of the strongest determinants of microbial composition and microbial metabolic activity. DI-GM was adapted from a literature-based index and has not been validated against gut microbiota composition in this population. Our finding that DI-GM was associated with gastric cancer risk (OR: 0.25 for highest vs. lowest tertile) should be considered exploratory pending validation studies linking DI-GM to microbial metagenomic data (29). Participants with higher DI-GM scores demonstrated substantially lower gastric cancer risk, supporting the hypothesis that microbiota-oriented dietary patterns were associated with lower odds of gastric cancer in the present study. Evidence specifically evaluating microbiota-oriented dietary indices in gastric cancer remains limited; however, emerging studies support a role of microbial dysbiosis and impaired gastric microbial ecology in gastric carcinogenesis.

Interestingly, among all evaluated biomarkers, DI-GM showed significant associations primarily with zonulin among gastric cancer cases. Zonulin is an important regulator of intestinal epithelial tight-junction integrity, and elevated concentrations may reflect increased intestinal permeability and compromised mucosal barrier function (30). Altered intestinal permeability may facilitate microbial translocation, endotoxemia, and chronic immune activation, all of which may contribute to carcinogenic processes (31, 32). The observed associations between DI-GM with zonulin therefore support the biological relevance of gut microbial pathways in gastric cancer pathophysiology (32). It is important to emphasize that the biomarkers measured in this study—including zonulin, LBP, TMAO, and calprotectin—reflect indirect indicators of gut microbial function, intestinal permeability, and microbial-host co-metabolism rather than direct measures of microbial community structure or compositional diversity. These biomarkers provide valuable insights into the downstream functional consequences of microbial activity and host–microbe interactions, but they cannot definitively characterize the specific bacterial taxa, relative abundances, or functional gene content of the gut microbiota. Direct microbiome sequencing (e.g., 16S rRNA gene amplicon sequencing or shotgun metagenomics) would be required to establish the compositional shifts, taxonomic signatures, and functional pathways directly linking dietary patterns to gut microbial ecology and gastric carcinogenesis. Given emerging evidence linking microbial dysbiosis, intestinal permeability, microbial metabolite imbalance, and immune dysregulation with gastric carcinogenesis, microbiota-oriented dietary indices may provide additional mechanistic insight beyond traditional inflammatory or antioxidant dietary measures. However, the mechanistic interpretation of the present findings should be tempered by the indirect nature of the biomarker-based approach employed.

The correlation analyses further demonstrated that DII generally exhibited positive relationships with inflammatory and oxidative biomarkers, whereas CDAI and DI-GM tended to show inverse correlations with these variables. Although many correlation coefficients were modest in magnitude, the overall directionality remained biologically coherent and supports the multidimensional interaction between dietary quality, systemic inflammation, oxidative stress, and gut dysfunction. Importantly, gastric carcinogenesis likely results from long-term cumulative biological exposure rather than isolated biomarker abnormalities; therefore, even moderate but consistent biomarker associations may be clinically meaningful. It should be noted that the biomarker-tertile comparisons were exploratory in nature and did not remain statistically significant after FDR correction. The modest correlation coefficients observed between dietary indices and biomarkers, while biologically coherent, should not be overinterpreted given the exploratory nature of these analyses and the absence of correction-surviving significance in the tertile-based comparisons. Consequently, these findings are best interpreted as hypothesis-generating rather than confirmatory. The principal confirmatory evidence from this study rests on the robust, pre-specified associations between the three dietary indices and gastric cancer risk.

The LASSO regression analysis provided additional insight into variables with the greatest discriminatory importance for gastric cancer. CRP, IL-6, TNF-α, zonulin, DII, NLR, and MDA demonstrated the strongest positive coefficients, emphasizing the potential importance of inflammatory activation, intestinal barrier dysfunction, and oxidative stress in disease discrimination. In contrast, CDAI, DI-GM, and TAC demonstrated negative coefficients, further supporting their potential protective biological roles. These findings collectively suggest that gastric cancer risk may involve a complex interaction among dietary inflammatory potential, antioxidant defense capacity, microbial metabolism, and intestinal permeability.

ROC analyses demonstrated moderate discriminatory performance for DII (AUC = 0.712), CDAI (AUC = 0.688), and DI-GM (AUC = 0.631). These AUC values indicate that none of the dietary indices alone possesses sufficient discriminative ability for clinical diagnostic or individual risk prediction purposes. In clinical settings, an AUC below 0.75 is generally considered inadequate for standalone diagnostic or prognostic use. However, the moderate performance suggests that these indices could contribute meaningfully to multifactorial risk stratification models when combined with established clinical risk factors (e.g., *H. pylori* status, family history, age, and lifestyle factors), microbial markers, and molecular biomarkers. From a public health perspective, these dietary indices may have greater utility in population-level screening and dietary intervention targeting than in individualized clinical risk prediction. The observed AUC values support the potential use of these indices as screening tools to identify high-risk populations for targeted dietary modification or lifestyle interventions, rather than as diagnostic instruments. Future studies integrating dietary indices with metabolomic, microbiota, and inflammatory biomarkers may improve the discriminatory performance of risk prediction algorithms and enable more precise identification of high-risk individuals for targeted prevention strategies.

The stratified analyses according to histological subtype, anatomical location, TNM stage, and metastatic status represent another important strength of the present study. Associations between dietary indices and gastric cancer remained generally consistent across tumor subgroups, suggesting that inflammatory, antioxidant, and microbiota-related dietary mechanisms may influence gastric carcinogenesis broadly rather than being restricted to specific tumor phenotypes. The stronger associations observed in advanced-stage and metastatic disease may suggest that unfavorable dietary exposures are associated with more aggressive disease phenotypes, systemic inflammation, and disease aggressiveness. Nevertheless, interaction analyses were not statistically significant, suggesting relative stability of associations across tumor characteristics.

Several methodological strengths should be acknowledged. The present study included a relatively large sample size with equal numbers of cases and controls, extensive adjustment for major confounders, comprehensive dietary assessment, and simultaneous evaluation of multiple biologically relevant dietary indices. Furthermore, we incorporated a broad panel of inflammatory, oxidative stress, intestinal permeability, and microbiota-related biomarkers, allowing multidimensional exploration of potential mechanisms linking diet and gastric cancer. The integration of correlation analyses, LASSO regression, ROC analyses, and tumor-stratified models additionally enhanced the analytical depth of the study.

However, several limitations should also be considered. First, the case–control design precludes definitive causal inference, and reverse causation cannot be entirely excluded. Individuals with undiagnosed gastric cancer may have altered their dietary habits due to early symptoms such as early satiety, dysphagia, nausea, or anorexia, potentially biasing estimates of the diet–cancer association. Although sensitivity analyses excluding participants with gastrointestinal symptoms (*n* = 237 cases, 49.4%) yielded consistent results (Supplementary Table S2), residual reverse causation cannot be fully ruled out. Furthermore, the retrospective assessment of dietary intake using FFQ methodology is subject to recall bias, and differential recall between cases and controls remains a concern. Second, dietary intake was assessed using FFQ methodology and therefore remains subject to recall bias and measurement error despite the use of validated instruments. Third, residual confounding from unmeasured variables cannot be completely excluded. Fourth, although multiple biomarkers were evaluated, direct gut microbiome sequencing and metagenomic profiling were not performed; therefore, microbial composition could not be directly characterized. Fifth, although we evaluated a comprehensive panel of gut associated biomarkers including zonulin, LBP, TMAO, and fecal calprotectin, direct gut microbiome sequencing or metagenomic profiling using stool microbiota was not performed. These biomarkers provide indirect insights into intestinal permeability, microbial metabolic activity, and endotoxin exposure, but they cannot substitute for direct characterization of microbial composition, diversity, or functional capacity. Future studies incorporating 16S rRNA sequencing or shotgun metagenomics are needed to elucidate the specific microbial taxa and functional pathways linking diet to gastric carcinogenesis. Given the large number of biomarker and subgroup analyses performed, the possibility of type I error due to multiple testing cannot be excluded. Finally, the study population was recruited from a limited geographic setting, which may reduce generalizability to other populations with different dietary habits, microbial environments, or genetic backgrounds.

## Conclusion

Overall, the present findings suggest that pro-inflammatory dietary patterns are associated with greater odds of gastric cancer, whereas antioxidant-rich and microbiota-supportive dietary patterns show inverse associations. These associations appear to involve multiple interconnected biological pathways including systemic inflammation, oxidative stress, intestinal permeability, and microbial metabolite regulation. Future prospective cohort studies with repeated dietary assessments, direct gut microbiome sequencing (e.g., 16S rRNA or shotgun metagenomics), metabolomic profiling, and mechanistic investigations (including animal models and intervention trials) are warranted to clarify causal pathways and determine whether targeted dietary modifications can beneficially modulate inflammatory and microbial pathways involved in gastric carcinogenesis. Because of the case–control design, reverse causation cannot be excluded; preclinical symptoms or differential recall may have influenced dietary reporting. Prospective studies are therefore needed to establish temporal relationships and strengthen causal inference.

## Data Availability

The datasets supporting the conclusions of this article will be made available by the corresponding author, upon reasonable request.
