# Supplementary material for: Inflammatory, antioxidant, and gut microbiota–related dietary indices and gastric cancer risk: a case–control study
Source: Front Nutr. 2026 Sep 16;13:1893032. doi: 10.3389/fnut.2026.1893032 (PMC13623746; doi:10.3389/fnut.2026.1893032)
Supplement: Supplementary file 1 [file Supplementary_file_1.docx]

| **STROBE Statement—Checklist of items that should be included in reports of case-control studies** | | | |
| --- | --- | --- | --- |
| **Section / Topic** | **Item No.** | **STROBE recommendation** | **Where addressed in manuscript** |
| Title and abstract | 1a | Indicate the study’s design with a commonly used term in the title or the abstract | Title and Abstract, p. 1 |
|  | 1b | Provide an informative and balanced summary of what was done and what was found | Abstract, p. 1 |
| Introduction – Background/rationale | 2 | Explain the scientific background and rationale for the investigation | Introduction, pp. 2–3 |
| Introduction – Objectives | 3 | State specific objectives, including any prespecified hypotheses | Introduction, p. 3 |
| Methods – Study design | 4 | Present key elements of study design early in the paper | Methods: Study design and participants, p. 3 |
| Methods – Setting | 5 | Describe the setting, locations, and relevant dates, including recruitment and data collection periods | Methods: Study design and participants, p. 3; Sample size, p. 3 |
| Methods – Participants | 6 | Give eligibility criteria, sources and methods of case ascertainment and control selection, and rationale for selection | Methods: Study design and participants; Sample size, pp. 3–4 |
| Methods – Participants | 6 | For matched studies, give matching criteria and number of controls per case | Age and sex frequency matching described in Sample size, p. 4 |
| Methods – Variables | 7 | Clearly define all outcomes, exposures, predictors, potential confounders, and effect modifiers | Clinical and tumor characteristics; Dietary assessment; Dietary indices; Laboratory and biomarker assessments, pp. 4–5 |
| Methods – Data sources/measurement | 8 | For each variable of interest, give sources and details of methods of assessment | Dietary assessment; Dietary indices; Laboratory and biomarker assessments, pp. 5–6 |
| Methods – Bias | 9 | Describe efforts to address potential sources of bias | Study design and participants; Dietary assessment; Statistical analysis; Discussion, pp. 4–6 and 9–10 |
| Methods – Study size | 10 | Explain how the study size was arrived at | Sample size, p. 3 |
| Methods – Quantitative variables | 11 | Explain how quantitative variables were handled in analyses; describe categorisation and cut-points if applicable | Dietary indices; Statistical analysis, pp. 5–6 |
| Methods – Statistical methods | 12a | Describe all statistical methods, including those used to control for confounding | Statistical analysis, p. 6 |
|  | 12b | Describe methods used to examine subgroups and interactions | Statistical analysis, p. 6; Stratified analyses, p. 8 |
|  | 12c | Explain how missing data were addressed | Study participants, p. 4; exclusions due to incomplete FFQ data are described |
|  | 12d | If applicable, describe analytical methods taking account of sampling strategy | Not applicable / not specifically used |
|  | 12e | Describe any sensitivity analyses | Statistical analysis; Results, pp. 6–7 |
| Results – Participants | 13a | Report numbers of individuals at each stage of study | Sample size; Figure 1, pp. 3–4 |
|  | 13b | Give reasons for non-participation at each stage | Sample size, p. 4 |
|  | 13c | Consider use of a flow diagram | Figure 1: Study flowchart, p. 4 |
| Results – Descriptive data | 14a | Give characteristics of study participants and information on exposures and potential confounders | Table 1, p. 7 |
|  | 14b | Indicate number of participants with missing data for each variable of interest | Missing FFQ data and exclusions described in Sample size, p. 4 |
|  | 14c | Summarise follow-up time, if applicable | Not applicable to this case–control study |
| Results – Outcome data | 15 | Report numbers of outcome events or summary measures | Tables 1–6; Results, pp. 7–12 |
| Results – Main results | 16a | Give unadjusted estimates and, if applicable, confounder-adjusted estimates and precision | Table 4, p. 9 |
|  | 16b | Report category boundaries when continuous variables were categorised | Dietary indices, p. 5; Table 4, p. 9 |
|  | 16c | Translate relative risk estimates into absolute risk if relevant | Not applicable; odds ratios were used for the case–control analysis |
| Results – Other analyses | 17 | Report other analyses done, e.g., subgroup analyses, interactions, sensitivity analyses | Stratified analyses; Sensitivity analyses, pp. 7–8 |
| Discussion – Key results | 18 | Summarise key results with reference to study objectives | Discussion, p. 9 |
| Discussion – Limitations | 19 | Discuss limitations, including potential sources of bias and imprecision | Discussion, pp. 9–13 |
| Discussion – Interpretation | 20 | Give a cautious overall interpretation considering objectives, limitations, multiplicity of analyses, and other relevant evidence | Discussion, pp. 9–13 |
| Discussion – Generalisability | 21 | Discuss the generalisability/external validity of the study results | Discussion, pp. 12–13 |
| Other information – Funding | 22 | Give the source of funding and role of funders | Funding, p. 13 |
