# Supplementary material for: Inflammatory, antioxidant, and gut microbiota–related dietary indices and gastric cancer risk: a case–control study
Source: Front Nutr. 2026 Sep 16;13:1893032. doi: 10.3389/fnut.2026.1893032 (PMC13623746; doi:10.3389/fnut.2026.1893032)
Supplement: Supplementary file 2 [file Table_1.docx]

**Supplementary Table 1. Biomarker Levels Across Tertiles of Dietary Indices Stratified by Gastric Cancer Status**

| **A. Dietary Inflammatory Index (DII)** | | | | | | | | |
| --- | --- | --- | --- | --- | --- | --- | --- | --- |
| **Biomarker** | **Controls T1 (n=226)** | **Controls T2 (n=163)** | **Controls T3 (n=91)** | **P-value** | **Cases T1 (n=94)** | **Cases T2 (n=157)** | **Cases T3 (n=229)** | **P-value** |
| CRP (mg/L) | 5.77 ± 2.70 | 5.58 ± 2.84 | 5.49 ± 3.06 | 0.670 | 8.69 ± 2.84 | 8.91 ± 2.71 | 8.66 ± 2.56 | 0.646 |
| IL-6 (pg/mL) | 7.36 ± 3.75 | 8.30 ± 4.14 | 8.83 ± 3.85 | 0.011 | 11.91 ± 4.12 | 12.07 ± 3.64 | 11.51 ± 4.08 | 0.368 |
| TNF-α (pg/mL) | 11.27 ± 4.05 | 10.99 ± 3.95 | 10.87 ± 4.43 | 0.672 | 14.44 ± 4.27 | 13.69 ± 4.04 | 14.42 ± 4.03 | 0.183 |
| Albumin (g/dL) | 3.97 ± 0.48 | 3.97 ± 0.50 | 4.01 ± 0.55 | 0.788 | 4.04 ± 0.52 | 3.94 ± 0.51 | 3.98 ± 0.50 | 0.372 |
| NLR | 2.80 ± 1.22 | 2.83 ± 1.23 | 2.73 ± 1.30 | 0.819 | 3.93 ± 1.20 | 3.65 ± 1.22 | 3.85 ± 1.38 | 0.199 |
| RDW (%) | 13.52 ± 1.15 | 13.45 ± 1.23 | 13.60 ± 1.17 | 0.609 | 13.57 ± 1.12 | 13.49 ± 1.10 | 13.64 ± 1.19 | 0.477 |
| MDA (µmol/L) | 3.87 ± 1.18 | 3.76 ± 1.31 | 3.79 ± 1.34 | 0.697 | 4.71 ± 1.00 | 4.86 ± 1.16 | 4.74 ± 1.30 | 0.564 |
| TAC (mmol/L) | 1.30 ± 0.30 | 1.33 ± 0.29 | 1.29 ± 0.31 | 0.520 | 1.14 ± 0.33 | 1.12 ± 0.30 | 1.08 ± 0.31 | 0.297 |
| Zonulin (ng/mL) | 47.55 ± 11.20 | 48.65 ± 13.26 | 48.50 ± 12.28 | 0.636 | 56.90 ± 12.19 | 56.30 ± 11.91 | 56.25 ± 11.80 | 0.897 |
| 8-OHdG (ng/mL) | 6.01 ± 1.77 | 5.93 ± 1.86 | 5.74 ± 2.18 | 0.527 | 6.23 ± 1.82 | 5.86 ± 1.91 | 5.99 ± 1.97 | 0.351 |
| LBP (µg/mL) | 18.66 ± 6.22 | 17.25 ± 6.40 | 18.13 ± 6.32 | 0.095 | 17.88 ± 6.23 | 18.37 ± 5.33 | 18.33 ± 6.13 | 0.782 |
| TMAO (µmol/L) | 4.46 ± 1.50 | 4.53 ± 1.53 | 4.56 ± 1.56 | 0.830 | 4.51 ± 1.33 | 4.50 ± 1.46 | 4.58 ± 1.47 | 0.830 |
| Fecal calprotectin (µg/g) | 54.64 ± 18.26 | 55.10 ± 17.72 | 51.21 ± 16.28 | 0.208 | 56.61 ± 20.64 | 53.69 ± 18.95 | 54.24 ± 18.95 | 0.486 |

**B. Composite Dietary Antioxidant Index (CDAI)**

| **Biomarker** | **Controls T1 (n=98)** | **Controls T2 (n=163)** | **Controls T3 (n=219)** | **P-value** | **Cases T1 (n=222)** | **Cases T2 (n=157)** | **Cases T3 (n=101)** | **P-value** |
| --- | --- | --- | --- | --- | --- | --- | --- | --- |
| CRP (mg/L) | 5.46 ± 2.72 | 5.60 ± 2.78 | 5.77 ± 2.89 | 0.652 | 8.93 ± 2.65 | 8.68 ± 2.79 | 8.47 ± 2.49 | 0.330 |
| IL-6 (pg/mL) | 9.11 ± 3.56 | 8.09 ± 4.15 | 8.26 ± 3.96 | 0.113 | 11.45 ± 3.90 | 12.37 ± 3.86 | 11.55 ± 4.12 | 0.067 |
| TNF-α (pg/mL) | 10.84 ± 4.27 | 11.06 ± 4.19 | 11.25 ± 3.94 | 0.708 | 14.11 ± 4.19 | 14.17 ± 4.04 | 14.37 ± 3.96 | 0.867 |
| Albumin (g/dL) | 4.00 ± 0.53 | 3.96 ± 0.47 | 3.99 ± 0.51 | 0.781 | 3.98 ± 0.49 | 4.00 ± 0.52 | 3.95 ± 0.52 | 0.762 |
| NLR | 2.78 ± 1.20 | 2.89 ± 1.20 | 2.73 ± 1.28 | 0.459 | 3.70 ± 1.26 | 3.92 ± 1.44 | 3.83 ± 1.13 | 0.276 |
| RDW (%) | 13.47 ± 1.28 | 13.57 ± 1.16 | 13.49 ± 1.15 | 0.735 | 13.52 ± 1.18 | 13.57 ± 1.14 | 13.72 ± 1.11 | 0.346 |
| MDA (µmol/L) | 3.89 ± 1.23 | 3.84 ± 1.26 | 3.77 ± 1.26 | 0.722 | 4.84 ± 1.16 | 4.74 ± 1.26 | 4.68 ± 1.19 | 0.501 |
| TAC (mmol/L) | 1.35 ± 0.34 | 1.31 ± 0.28 | 1.28 ± 0.29 | 0.183 | 1.11 ± 0.33 | 1.11 ± 0.30 | 1.09 ± 0.30 | 0.919 |
| Zonulin (ng/mL) | 48.89 ± 11.74 | 47.97 ± 12.36 | 47.84 ± 12.15 | 0.765 | 56.47 ± 12.04 | 55.90 ± 12.17 | 56.99 ± 11.19 | 0.766 |
| 8-OHdG (ng/mL) | 5.91 ± 1.76 | 5.92 ± 1.98 | 5.95 ± 1.86 | 0.985 | 6.06 ± 1.83 | 5.91 ± 1.99 | 5.98 ± 2.04 | 0.745 |
| LBP (µg/mL) | 18.38 ± 5.99 | 17.70 ± 6.48 | 18.23 ± 6.36 | 0.628 | 18.21 ± 5.92 | 18.17 ± 5.65 | 18.50 ± 6.24 | 0.897 |
| TMAO (µmol/L) | 4.80 ± 1.52 | 4.37 ± 1.50 | 4.46 ± 1.52 | 0.083 | 4.44 ± 1.44 | 4.69 ± 1.45 | 4.53 ± 1.42 | 0.250 |
| Fecal calprotectin (µg/g) | 53.20 ± 18.31 | 55.90 ± 16.43 | 53.26 ± 18.39 | 0.299 | 54.56 ± 19.24 | 53.75 ± 20.03 | 55.65 ± 18.27 | 0.742 |

**C. Dietary Index for Gut Microbiota (DI-GM)**

| **Biomarker** | **Controls T1 (n=114)** | **Controls T2 (n=147)** | **Controls T3 (n=219)** | **P-value** | **Cases T1 (n=206)** | **Cases T2 (n=174)** | **Cases T3 (n=100)** | **P-value** |
| --- | --- | --- | --- | --- | --- | --- | --- | --- |
| CRP (mg/L) | 5.50 ± 2.76 | 5.90 ± 2.94 | 5.56 ± 2.76 | 0.424 | 8.46 ± 2.61 | 8.96 ± 2.53 | 8.99 ± 2.96 | 0.119 |
| IL-6 (pg/mL) | 8.04 ± 3.94 | 8.16 ± 3.83 | 8.69 ± 4.05 | 0.266 | 11.84 ± 3.93 | 11.67 ± 4.17 | 11.79 ± 3.61 | 0.914 |
| TNF-α (pg/mL) | 11.37 ± 4.33 | 10.60 ± 4.22 | 11.30 ± 3.85 | 0.204 | 14.20 ± 4.18 | 14.30 ± 4.02 | 13.96 ± 4.05 | 0.804 |
| Albumin (g/dL) | 3.96 ± 0.52 | 3.96 ± 0.48 | 4.00 ± 0.50 | 0.714 | 3.96 ± 0.51 | 4.03 ± 0.50 | 3.92 ± 0.50 | 0.179 |
| NLR | 2.74 ± 1.25 | 2.90 ± 1.16 | 2.76 ± 1.28 | 0.480 | 3.78 ± 1.37 | 3.77 ± 1.22 | 3.90 ± 1.30 | 0.691 |
| RDW (%) | 13.52 ± 1.26 | 13.56 ± 1.11 | 13.48 ± 1.19 | 0.834 | 13.61 ± 1.12 | 13.59 ± 1.21 | 13.49 ± 1.11 | 0.700 |
| MDA (µmol/L) | 3.81 ± 1.22 | 3.74 ± 1.31 | 3.88 ± 1.24 | 0.617 | 4.80 ± 1.24 | 4.75 ± 1.14 | 4.76 ± 1.23 | 0.936 |
| TAC (mmol/L) | 1.26 ± 0.31 | 1.33 ± 0.31 | 1.31 ± 0.29 | 0.146 | 1.10 ± 0.32 | 1.10 ± 0.31 | 1.11 ± 0.32 | 0.945 |
| Zonulin (ng/mL) | 49.82 ± 12.58 | 47.41 ± 11.64 | 47.67 ± 12.17 | 0.217 | 54.96 ± 11.26 | 58.33 ± 11.68 | 55.97 ± 13.11 | **0.021** |
| 8-OHdG (ng/mL) | 5.72 ± 1.83 | 5.95 ± 1.87 | 6.03 ± 1.92 | 0.372 | 5.76 ± 1.89 | 6.12 ± 1.96 | 6.24 ± 1.91 | 0.071 |
| LBP (µg/mL) | 18.03 ± 5.99 | 17.94 ± 5.94 | 18.21 ± 6.74 | 0.917 | 17.62 ± 6.00 | 18.42 ± 5.58 | 19.28 ± 6.07 | 0.062 |
| TMAO (µmol/L) | 4.58 ± 1.64 | 4.56 ± 1.44 | 4.42 ± 1.51 | 0.560 | 4.62 ± 1.41 | 4.38 ± 1.45 | 4.66 ± 1.47 | 0.174 |
| Fecal calprotectin (µg/g) | 55.61 ± 18.63 | 53.66 ± 18.73 | 53.71 ± 16.58 | 0.600 | 56.40 ± 19.39 | 54.05 ± 19.13 | 51.47 ± 19.03 | 0.102 |

*Abbreviations:* DII, Dietary Inflammatory Index; CDAI, Composite Dietary Antioxidant Index; DI-GM, Dietary Index for Gut Microbiota; T1, lowest tertile; T2, middle tertile; T3, highest tertile; CRP, C-reactive protein; IL-6, interleukin-6; TNF-α, tumor necrosis factor-α; NLR, neutrophil-to-lymphocyte ratio; RDW, red cell distribution width; MDA, malondialdehyde; TAC, total antioxidant capacity; 8-OHdG, 8-hydroxy-2′-deoxyguanosine; LBP, lipopolysaccharide-binding protein; TMAO, trimethylamine N-oxide.

*Values are mean ± SD. P-values were derived from one-way ANOVA across tertiles. Bold values indicate statistical significance (P < 0.05).*

**Supplementary Table 2. Sensitivity Analysis Excluding Participants with Gastrointestinal Symptoms**

| Dietary Index | Comparison | Main Analysis (n=960) | Sensitivity Analysis (n=724) * |
| --- | --- | --- | --- |
| **DII** |  |  |  |
|  | T2 vs. T1 | 2.33 (1.68–3.24) | 1.87 (1.20–2.91) |
|  | T3 vs. T1 | 6.21 (4.39–8.79) | 5.61 (3.60–8.73) |
| **CDAI** |  |  |  |
|  | T2 vs. T1 | 0.42 (0.30–0.58) | 0.39 (0.26–0.59) |
|  | T3 vs. T1 | 0.20 (0.14–0.28) | 0.21 (0.14–0.33) |
| **DI-GM** |  |  |  |
|  | T2 vs. T1 | 0.67 (0.48–0.92) | 0.84 (0.55–1.26) |
|  | T3 vs. T1 | 0.25 (0.18–0.35) | 0.33 (0.21–0.52) |

Abbreviations: DII, Dietary Inflammatory Index; CDAI, Composite Dietary Antioxidant Index; DI-GM, Dietary Index for Gut Microbiota; OR, odds ratio; CI, confidence interval.

*Values are presented as odds ratios (95% confidence intervals) from multivariable logistic regression models (Model 2) adjusted for age, sex, BMI, smoking pack-years, alcohol intake, physical activity, H. pylori status, total energy intake, education level, PPI/H2 blocker use, socioeconomic status, and family history of gastric cancer.

†Sensitivity analysis excluded 237 cases (49.4%) who reported gastrointestinal symptoms (early satiety, dysphagia, nausea, or anorexia) that may have led to dietary modifications prior to diagnosis. The remaining sample comprised 243 cases and 481 controls (n=724).
